# Bridging Primary and Specialist Care in Atopic Dermatitis: Outcomes of an Interregional Referral Protocol in Portugal

**DOI:** 10.7759/cureus.99541

**Published:** 2025-12-18

**Authors:** Rita Branco Vargas, Tomás Costa, Teresa Leitão, Pedro Farinha, Miguel Peliteiro, Bruno Duarte, Cátia Santos

**Affiliations:** 1 General Practice, Unidade Local (ULS) de Saúde do Alentejo Central, Évora, PRT; 2 Dermatology, Unidade Local (ULS) de Saúde Amadora-Sintra, Amadora, PRT; 3 Family Medicine, Unidade Local de Saúde (ULS) do Alentejo Central, Évora, PRT; 4 Dermatology, Unidade Local de Saúde (ULS) de São José, Lisbon, PRT; 5 Neuroradiology, Unidade Local de Saúde (ULS) de São João, Porto, PRT

**Keywords:** atopic dermatits, clinical dermatology, inter-professional practice, patient-centred care, primary care medicine

## Abstract

Atopic dermatitis (AD) is a chronic inflammatory skin disease with a significant impact on quality of life and healthcare systems. In Portugal, access to specialist care remains limited, particularly for patients requiring advanced therapies available only in hospital settings. This study aimed to implement and evaluate a structured referral protocol between primary and hospital dermatology services to improve AD management.

Between April 2024 and February 2025, adult patients (≥18 years) coded with AD were identified at the USF Planície primary care center and assessed using a structured telephone questionnaire evaluating disease severity (Patient-Oriented Eczema Measure (POEM)), pruritus (Itch Numeric Rating Scale (INRS)), and sleep disturbance (Sleep Numeric Rating Scale (SNRS)).

Of 213 identified patients, 119 (55.8%) were excluded - 94 (44.1%) could not be contacted; 19 (8.9%) denied the diagnosis; and 6 (2.8%) refused to participate - and 94 (44.1%) completed the assessment. Among these patients, 74 (78.7%) had mild or well-controlled disease, whereas 21 (22.3%) presented with moderate-to-severe AD. Patients with moderate-to-severe POEM showed a higher disease burden, with INRS ≥ 5 in 17 patients (85.0%), SNRS ≥ 5 in 5 patients (25.0%), and involvement of high-impact areas in 15 patients (75.0%), whereas in mild POEM, most patients had INRS < 5 (71, 95.9%), SNRS < 5 (74, 100%), and limited involvement of high-impact areas (16, 21.6%).

This protocol demonstrated feasibility and clinical relevance, improving patient stratification and facilitating timely referral for specialist evaluation.

## Introduction

Atopic dermatitis (AD) is a chronic, relapsing inflammatory skin disease characterized by pruritus and eczematous lesions, affecting both children and adults worldwide [1]. Recent global epidemiological analyses estimate that AD affects approximately 204 million individuals globally [2]. However, significant heterogeneity persists in case definitions and severity assessments across regions, complicating direct comparisons and highlighting persistent gaps in global burden data [3].

Consistent with global patterns, patients in Portugal frequently experience diagnostic delays, high rates of allergic comorbidities, and considerable psychosocial distress, including anxiety, sleep disturbance, and stigmatization [4,5]. Disease severity is closely associated with reduced perceived health and increased suffering, particularly among those with moderate-to-severe AD [4]. The economic burden is substantial, encompassing direct medical costs, absenteeism, and out-of-pocket expenditures, with annual costs in Portugal alone estimated to exceed €1 billion [6].

Despite major therapeutic advances, including new biologic agents and targeted immunomodulators, many patients remain undertreated or lack access to effective therapies [4,7]. This challenge is particularly pronounced in Portugal, where advanced systemic treatments are available exclusively through hospital-based dermatology services in the public National Health System (NHS). This exclusivity underscores the urgent need for optimized referral pathways to ensure equitable access to specialized care.

However, structural barriers persist within this public NHS, which is organized into Local Health Units (LHS). A critical gap exists for patients at USF Planície (ULS Alentejo Central), as their reference hospital in Évora lacks a Dermatology Department, hindering appropriate referrals. In contrast, ULS Lisboa Central operates a specialized AD clinic designed to function as a national referral centre.

To address this specific inequality, the primary objective of this study was to facilitate the appropriate referral of patients from primary care to dermatology by leveraging the existing infrastructure in Lisbon. To achieve this, a structured referral protocol was developed to establish a standardized pathway between the two public institutions, ensuring that patients in Alentejo Central receive the specialized secondary care currently inaccessible locally.

Secondary objectives included characterizing adult patients with AD managed in primary care, strengthening epidemiological data for the Portuguese population, and assessing the impact of the disease on quality of life.

## Materials and methods

This was a prospective implementation study carried out between April 2024 and February 2025 at USF Planície, part of the ULS Alentejo Central, in collaboration with the Hospital dos Capuchos (ULS São José), Portugal - the project aimed to standardize the identification and referral of adults with AD in the primary care setting.

**Figure 1:**
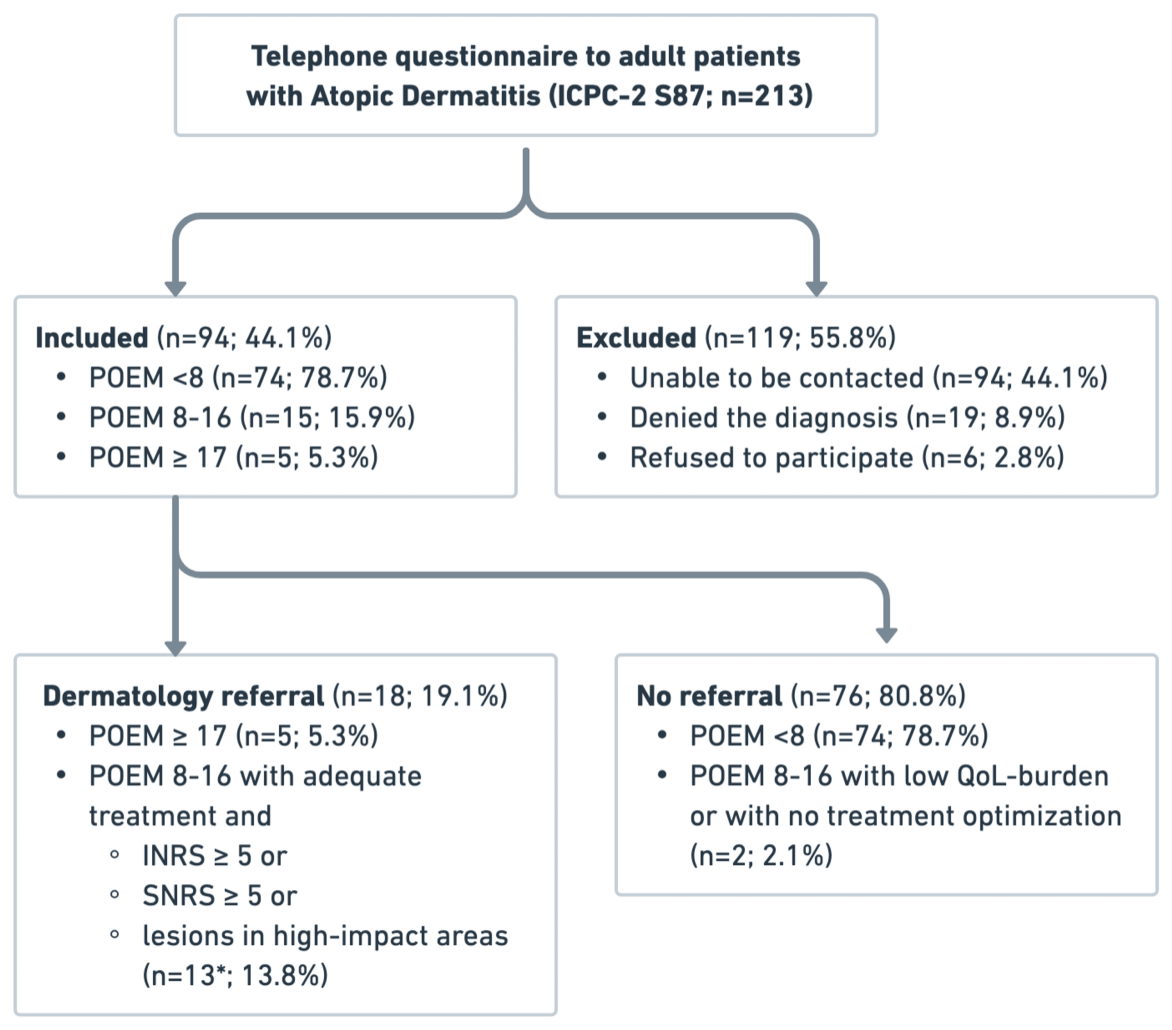
Cohort flowchart. ICPC-2, International Classification of Primary Care, 2nd edition; POEM, Patient-Oriented Eczema Measure; INRS, Itch Numeric Rating Scale; SNRS, Sleep Numeric Rating Scale; QoL, quality of life *One patient (POEM 9, hand lesions, INRS 10) declined referral due to unwillingness to travel to another city.

The study population (Figure 1) included all adult patients aged 18 years or older with a diagnosis of “Atopic dermatitis/eczema” (ICPC-2 code S87) previously coded by Family Physicians of USF Planície during routine consultations, consistent with the mandatory registration of clinical history required within the Portuguese NHS electronic system. Eligible participants were those with a confirmed diagnosis of AD who consented to participate. Patients who refused participation, could not be reached after multiple contact attempts, or denied having the condition were excluded from the analysis.

This implementation study was based on a telephone questionnaire (Table 1) administered by physicians at USF Planície, who are also co-authors of this work. To ensure consistency, the medical team underwent specific training on the administration of the questionnaire and adherence to the full study protocol. The questionnaire included verification of the physician’s identity, as well as confirmation of the patient’s identification and diagnosis of AD. It also explained the project and emphasized that participation in the study was entirely voluntary, with no consequences in the event of refusal.

**Table 1:** Telephone interview guide for physicians. POEM, Patient-Oriented Eczema Measure; INRS, Itch Numeric Rating Scale; SNRS, Sleep Numeric Rating Scale

Section	Interview script
1. Eligibility	Identification of the physician; confirmation of the patient’s full name; confirmation of the previous diagnosis of Atopic Dermatitis.	
	Explanation of the project’s objective – to improve the treatment and referral of patients with atopic dermatitis.
	Explanation of the voluntary nature of the project and that refusal to participate in the study has no negative consequences.
	Obtaining consent to participate in the study.
2. Clinical Assessment	
2.1 Disease Severity (POEM)	"Over the last week, on how many days has your skin been itchy because of eczema?"
	"Over the last week, on how many nights has your sleep been disturbed because of eczema?"
	"Over the last week, on how many days has your skin been bleeding because of eczema?"
	"Over the last week, on how many days has your skin been weeping or oozing clear fluid because of eczema?"
	"Over the last week, on how many days has your skin been cracked because of eczema?"
	"Over the last week, on how many days has your skin been flaking off because of eczema?"
	"Over the last week, on how many days has your skin felt dry or rough because of eczema?"
2.2 Pruritus and Sleep Impact (INRS; SNRS)	"On a scale from 0 to 10, how intense has your itching been during the last week?"
	"On a scale from 0 to 10, how much has eczema disturbed your sleep?"
2.3 Lesion Distribution	"Do you currently have eczema lesions on your face, hands, or genital region?"
2.4 Treatment Practices	"Do you apply emollients every day, even when your skin is clear?"
	"When you have active/recurrent eczema lesions, do you apply topical corticosteroids or calcineurin inhibitors?"
	"How do you usually apply these treatments (frequency, duration, and amount)?"

Participants who agreed to take part subsequently completed a clinical questionnaire, which assessed the severity of AD using the Patient-Oriented Eczema Measure (POEM) scale [8]; documented the distribution of lesions; and evaluated the impact on quality of life through the Itch Numeric Rating Scale (INRS) and the Sleep Numeric Rating Scale (SNRS) [9,10]. This standardized clinical protocol was developed and made available to the medical team using a digital clinical decision support system (Dioscope©, Lisbon, Portugal). The questionnaire further examined the adequacy of topical treatment, including daily application of emollients, use of topical corticosteroids or calcineurin inhibitors during acute eczema flares, and, in cases of sites with recurrent lesions, the proactive use of topical corticosteroids or calcineurin inhibitors. 

Referral criteria were defined to target patients with moderate-to-severe disease refractory to standard care, in alignment with established international recommendations [8-10]. Specifically, patients met the criteria if they presented with a POEM score ≥ 17, or POEM ≥ 8 with inadequate topical treatment plus significant burden (INRS ≥ 5, SNRS ≥ 5) or lesions in high-impact areas (face, hands, or genital region). Patients who did not meet these thresholds were to remain under follow-up in primary care.

Data were analyzed using IBM SPSS version 30.0 (IBM Corp., Armonk, NY). Associations between categorical variables were examined using Pearson’s chi-square or Fisher’s exact tests [11,12], supplemented by standardized residuals analysis to identify significant deviations [13]. Effect sizes were measured using the phi coefficient (Φ) [14,15]. All tests were two-tailed with significance defined as *P* < 0.05, following established recommendations for small-sample inference in clinical research [12,16].

## Results

A total of 213 adult patients coded with AD/eczema (ICPC-2 code S87) were identified in the electronic medical records of USF Planície. Of these, 94 individuals (44.1%) were successfully contacted and completed the structured telephone questionnaire, while 119 (55.9%) were excluded: 94 (44.1%) due to lack of response, 19 (8.9%) who denied the diagnosis upon re-contact, and 6 (2.8%) who refused to participate.

Among the 94 patients who completed the assessment, 74 (78.7%) had POEM scores below 8, indicating mild or well-controlled disease. The remaining patients were classified as having moderate disease (15, 16%) or severe disease (5, 5.3%).

Prevalence of high-impact areas, including the face, hands, or genital region, was 31 out of 94 participants (33%). Table 2 presents the association between POEM severity and the presence of a high-impact area. A chi-square test revealed a statistically significant association between the two variables, χ²(1) = 20.30, *P* < 0.001, indicating a large effect size (Φ = 0.47). Among participants with mild POEM scores (n = 74), the majority (n=58, 78.4%) were classified as not having a high-impact area disease, while 16 (21.6%) were classified as having one. Conversely, in the moderate-to-severe POEM group (n = 20), high-impact area involvement was observed in 15 (75%) of patients, with sparing in only 5 (25%). The standardized residuals indicated that mild cases were significantly overrepresented in the “no high-impact area” category (rᵢ = -2.3) and underrepresented in the “yes high-impact area” category, while the opposite pattern was observed for moderate-to-severe cases (rᵢ = 3.3). These findings suggest that higher POEM severity is strongly associated with a greater likelihood of involvement in high-impact anatomical regions. 

**Table 2:** Association between POEM severity and high-impact area. Results are presented as *n* (%). r_i_ = standardized Pearson residual. The association was assessed using the chi-square test (χ²(1) = 20.30, *P* < 0.001, φ = 0.47), and standardized residuals are shown when higher than +1.96 or lower than –1.96, indicating a statistically significant deviation from expected frequencies. Effect size was calculated using phi (Φ), following Cohen’s conventions for 0.1 (small), 0.3 (medium), and 0.5 (large) effects [14]. POEM, Patient-Oriented Eczema Measure

	POEM = Mild (*n* = 74)	POEM = Moderate to severe (*n* = 20)	
High-impact area = no	58 (78.4%)	5 (25%) (ri = -2.3)	
High-impact area = yes	16 (21.6%)	15 (75.0%) (ri = 3.3)	

Table 3 shows the association between POEM severity and INRS and SNRS prevalence. Among patients with mild POEM (*n* = 74), 71 (95.9%) had INRS < 5 and 3 (4.1%) had INRS ≥ 5, with standardized residuals indicating a significant deficit of mild cases in the higher INRS category (r_i_ = -3.2) and a corresponding deficit in the lower INRS category for moderate-to-severe POEM. Among patients with moderate-to-severe POEM (n = 20), 3 (15.0%) had INRS < 5 and 17 (85.0%) had INRS ≥ 5, with a large positive residual for INRS ≥ 5 (r_i_ = 6.2) indicating a significant excess of moderate-to-severe cases in the higher INRS category. The association between POEM severity and INRS was statistically significant (*P *< 0.001) with a strong effect size (Φ = 0.81). For SNRS, all patients with mild POEM had SNRS < 5 (74, 100%) and none had SNRS ≥ 5 (0, 0%) with residuals showing a significant deficit in high SNRS for mild cases (r_i_ = -2.0). Among moderate-to-severe POEM, 15 (75.0%) had SNRS < 5 (r_i _= -2.2) and 5 (25.0%) had SNRS ≥ 5 (r_i_ = 3.8), indicating a significant excess of moderate-to-severe cases with higher SNRS. The association between POEM severity and SNRS was statistically significant (*P *< 0.001) with a moderate effect size (Φ = 0.47). Overall, standardized residuals indicate that mild POEM cases are underrepresented in higher INRS and SNRS categories, while moderate-to-severe cases are overrepresented, supporting a strong relationship between POEM severity and both INRS and SNRS scores.

**Table 3:** Association of POEM severity with INRS and SNRS (0 vs. ≥ 1). Results are presented as *n* (%). Associations were assessed using Fisher’s exact test. r_i_ = standardized Pearson residual. Standardized residuals are shown when higher than +1.96 or lower than –1.96, indicating a statistically significant deviation from expected frequencies. Effect size was calculated using phi (Φ), following Cohen’s conventions for 0.1 (small), 0.3 (medium), and 0.5 (large) effects [14]. For INRS, *P* < 0.001, Φ = 0.81; for SNRS, *P* < 0.001, Φ = 0.47. POEM, Patient-Oriented Eczema Measure

	POEM = Mild (*n* = 74)	POEM = Moderate to severe (*n *= 20)
INRS < 5	71 (95.9%)	3 (15.0%) (r_i_ = -3.2)
INRS ≥ 5	3 (4.1%) (r_i_ = -3.2)	17 (85.0%) (ri = 6.2)
SNRS < 5	74 (100%)	15 (75.0%) (r_i _= -2.2)
SNRS ≥ 5	0 (0%) (r_i _= -2.0)	5 (25.0%) (r_i _= 3.8)

Regarding treatment patterns, the 25 patients on no topical therapy had a mean POEM score of 1.41, with 23 scoring < 8 and only two exceeding this threshold (scores: 9 and 12). The 36 patients using emollients exclusively all presented with controlled disease (POEM < 8; mean: 1.08). Conversely, patients on topical corticosteroids alone (*n* = 6) had a mean POEM of 9.66, with an equal split between controlled (*n* = 3) and uncontrolled (*n *= 3) disease. Finally, the cohort using combination therapy (emollients plus corticosteroids; *n *= 15) showed the highest disease burden (mean POEM: 11.0), with 12 patients scoring ≥ 8 and only 3 below this cutoff.

Regarding patient referral, 18 patients (19.1%) met the criteria for dermatology referral. Among these, 5 (27.8%) had POEM scores ≥17 and therefore fulfilled referral criteria by definition, while 13 presented with POEM scores ≥8 and either INRS ≥5, SNRS ≥5, or lesions in high-impact areas despite appropriate topical therapy, thus meeting the established criteria. Of these 13, 1 (7.7%) declined referral despite fulfilling clinical indications because he did not want to travel to a hospital in another city. In addition, 2 patients with POEM scores ≥8 were not receiving adequate topical treatment and therefore did not meet referral criteria.

## Discussion

According to the public database available on the Portuguese NHS portal [17], USF Planície provides care to 12,774 adult patients (≥18 years). Thus, with 213 patients coded with AD, the estimated prevalence is 1.6%. These data are concordant with other epidemiological evaluations for the adult population, but - as previously noted - limited by some heterogeneity in case definitions [3].

In this primary care cohort, over one in five patients (21.3%) presented with moderate-to-severe AD. These findings complement and extend previous epidemiological data indicating that approximately 70,000 individuals in Portugal live with moderate-to-severe AD, based on an estimated national prevalence of 360,000 cases (3.6% of the Portuguese population) [4].

These data gain particular relevance when analyzing the relationship between disease severity and quality-of-life impact. In this context, our study demonstrates that higher POEM severity is strongly associated with a greater likelihood of involvement of high-impact anatomical areas, and that POEM scores correlate closely with both INRS and SNRS indices.

Within the moderate-to-severe subgroup, 75% reported lesions in high-impact areas, and 85% had INRS scores above 5 - the predefined referral threshold - indicating a substantial pruritic burden. In contrast, sleep disturbance, defined as SNRS >5, was less common, affecting 25% of patients. These findings align with previous studies showing that pruritus is the most prominent and distressing symptom of AD, with mean INRS values consistently exceeding SNRS or other sleep-related measures in both clinical trials and real-world cohorts [18,19]. Although sleep impairment is a recognized component of disease burden, its severity scores are generally lower than itch scores and may not fully capture nocturnal symptom impact. Psychometric studies have demonstrated only moderate correlations between itch and sleep disturbance, indicating that these are related yet distinct domains [10,20]. Moreover, documented floor effects in sleep-related instruments suggest that some patients report minimal sleep disruption despite significant itch intensity, possibly due to habituation or under-recognition of impaired sleep quality [21].

With regard to treatment adherence, 26.6% of patients were not following the general skin care measures routinely recommended for AD management. These adherence rates are consistent with international studies employing objective methods such as electronic monitoring or pharmacy refill data, which report adherence to topical therapy ranging between 30% and 50%. Contributing factors include regimen complexity, fear of adverse effects-particularly corticosteroid phobia-limited patient education, and suboptimal physician-patient communication [22-24].

However, among the 15 patients with moderate disease, only 2 (13.3%) were not using appropriate topical treatment. While this may reflect increased awareness of treatment needs among patients with visible or high-impact lesions, it also highlights the limitations of AD management within primary care once therapeutic optimization is achieved. Despite persistent disease activity, many of these patients are not referred to dermatology.

This pattern mirrors European data showing that numerous patients with moderate-to-severe AD remain managed exclusively in primary care, contrary to guideline recommendations advocating specialist referral when the disease is uncontrolled or substantially impairs quality of life [7]. Although Portuguese data remain scarce, a recent national study reported that only 19% of patients with severe AD were receiving biologic therapy - available exclusively through hospital-based dermatology services - suggesting that most moderate-to-severe cases do not reach specialist care [4].

A distinctive aspect of this study was the implementation of an inter-regional referral protocol between USF Planície (Évora), a primary care unit lacking a dermatology department, and the dermatology team at ULS São José (Lisbon, approximately 130 km away). This initiative addressed structural barriers in referral pathways and represented an innovative inter-institutional collaboration within the Portuguese National Health Service. By establishing clear, objective referral criteria and facilitating direct communication between primary care and a tertiary hospital, this model reduced bureaucratic obstacles and ensured timely specialist evaluation for patients with uncontrolled disease.

Several limitations should be acknowledged. The study was conducted in a single primary care unit, which may limit conclusions to other regions or healthcare systems. The population served by USF Planície may differ in demographic or socioeconomic characteristics from those in more urbanized areas. Data collection relied on a structured telephone-based questionnaire, which, although efficient and well accepted, is subject to recall and perception bias. More than half of coded patients did not complete the questionnaire (non-response, refusal, or denial of diagnosis), which may bias severity estimates and limit generalizability. Additionally, this study did not include a follow-up to assess whether referral and subsequent dermatological consultation improved disease control or quality of life. Future research should therefore incorporate longitudinal evaluation to measure the impact of structured referral protocols on clinical outcomes, healthcare utilization, and patient satisfaction. Integration of digital tools for remote monitoring and automatic identification of patients meeting referral criteria could further streamline this process and support broader national implementation.

Finally, although validated instruments such as POEM, INRS, and SNRS were used, the interpretation of patient-reported outcomes may vary across cultural and linguistic contexts. Future multicenter studies involving larger and more diverse samples would be valuable to confirm these findings and refine referral thresholds appropriate for the Portuguese healthcare setting.

Despite these limitations, this study demonstrates the feasibility and clinical value of integrating standardized assessment and referral pathways for AD into routine primary care. The findings highlight the potential of data-driven protocols to enhance coordination between care levels, reduce diagnostic delays, and ensure equitable access to dermatology services for patients with inadequately controlled disease.

## Conclusions

This study highlights the significant burden of AD in primary care, where a notable proportion of patients present with moderate-to-severe disease requiring specialist management. The implementation of a structured referral protocol between primary and hospital dermatology services proved feasible and effective, facilitating proper access to specialized care. These findings support the integration of standardized, data-driven referral pathways within the Portuguese healthcare system to improve disease control, reduce delays, and ensure equitable access to advanced treatments.
